# The innate immune and systemic response in honey bees to a bacterial pathogen, *Paenibacillus larvae*

**DOI:** 10.1186/1471-2164-10-387

**Published:** 2009-08-21

**Authors:** Queenie WT Chan, Andony P Melathopoulos, Stephen F Pernal, Leonard J Foster

**Affiliations:** 1 Centre for High-Throughput Biology and Department of Biochemistry & Molecular Biology, University of British Columbia, Vancouver, BC, V6T 1Z4, Canada; 2 Agriculture and Agri-Food Canada, Beaverlodge Research Farm, PO Box 29, Beaverlodge, AB, T0H 0C0, Canada

## Abstract

**Background:**

There is a major paradox in our understanding of honey bee immunity: the high population density in a bee colony implies a high rate of disease transmission among individuals, yet bees are predicted to express only two-thirds as many immunity genes as solitary insects, e.g., mosquito or fruit fly. This suggests that the immune response in bees is subdued in favor of social immunity, yet some specific immune factors are up-regulated in response to infection. To explore the response to infection more broadly, we employ mass spectrometry-based proteomics in a quantitative analysis of honey bee larvae infected with the bacterium *Paenibacillus larvae*. Newly-eclosed bee larvae, in the second stage of their life cycle, are susceptible to this infection, but become progressively more resistant with age. We used this host-pathogen system to probe not only the role of the immune system in responding to a highly evolved infection, but also what other mechanisms might be employed in response to infection.

**Results:**

Using quantitative proteomics, we compared the hemolymph (insect blood) of five-day old healthy and infected honey bee larvae and found a strong up-regulation of some metabolic enzymes and chaperones, while royal jelly (food) and energy storage proteins were down-regulated. We also observed increased levels of the immune factors prophenoloxidase (proPO), lysozyme and the antimicrobial peptide hymenoptaecin. Furthermore, mass spectrometry evidence suggests that healthy larvae have significant levels of catalytically inactive proPO in the hemolymph that is proteolytically activated upon infection. Phenoloxidase (PO) enzyme activity was undetectable in one or two-day-old larvae and increased dramatically thereafter, paralleling very closely the age-related ability of larvae to resist infection.

**Conclusion:**

We propose a model for the host response to infection where energy stores and metabolic enzymes are regulated in concert with direct defensive measures, such as the massive enhancement of PO activity.

## Background

Honey bees, *Apis mellifera*, face a number of niche-specific pathogens such as the endospore-forming bacterium *Paenibacillus larvae*, the causative agent of American Foulbrood (AFB) [1]. Bees are only susceptible to *P. larvae *during the first 48 h following eclosion (egg hatching), in their first and second instar developmental stages. It remains unclear why larvae acquire immunity against *P. larvae *after the third instar, whereas the ingestion of merely 10 spores can cause systemic infection and death in the previous instars [2]. It was thought that *P. larvae *spores germinate in the larval midgut and enter the epithelium by phagocytosis [3,4] but recent data suggest that the bacteria follow a paracellular route to breach the epithelial wall [5]. The effectiveness of the antimicrobial peptide (AMP) defensin against *P. larvae *was documented in growth inhibition assays [6,7] using fractionated royal jelly (honey bee food). However, Evans *et al*. found no changes in defensin gene expression in larvae fed *P. larvae *spores and, paradoxically, that abaecin (another AMP) gene expression was greatest in newly eclosed larvae [8,9], the most susceptible stage. More recently, the same group showed that infection caused elevated expression of Toll-like receptor, MyD88 and IκB [10]. Thus, even though they do not always respond as expected, honey bees have all the components of an innate immune system. Here we explore the response of this system to a physiologically relevant infection in a natural setting.

To this end, hemolymph (arthropod blood) is well-suited for studying insect immunity; it is especially relevant in the case of *P. larvae *as the bacterium contacts hemolymph as soon as it breaches the gut epithelium. This fluid contains antimicrobial factors produced largely by the fat body and, to a lesser extent, hemocytes. These cells can also respond to infectious particles by phagocytosing them or by autolysis, which is part of an encapsulation pathway used to inhibit growth of microorganisms. Likewise, as the connective tissue responsible for transporting various molecules throughout the body, it is also optimally suited for monitoring systemic changes in other pathways. Previously, we have examined how hemolymph changes during normal larval development [11]. In that study we observed that most immune factors were not significantly altered during development, with only the AMP apismin and the monooxygenase prophenoloxidase showing any age-related changes in expression. Thus, based on our earlier work and that of others [8-12], we expected that a *P. larvae *infection should induce, in hemolymph, elevated levels of at least some AMPs, as well as other antibacterial enzymes such as lysozyme and prophenoloxidase. To address these predictions, we use mass spectrometry (MS)-based proteomics to measure changes in hemolymph protein levels in larvae challenged with *P. larvae*. Furthermore, we predict that a protein that is able to convey immunity to older larvae and adults must be expressed at extremely low levels in the susceptible early larval instars. Using a functional assay, we demonstrate how one potentially critical player in host defense, phenoloxidase (PO), correlates with larval resistance to infection.

## Results

### Different strains of *P. larvae *produce equivalent outcomes

**Figure 1:**
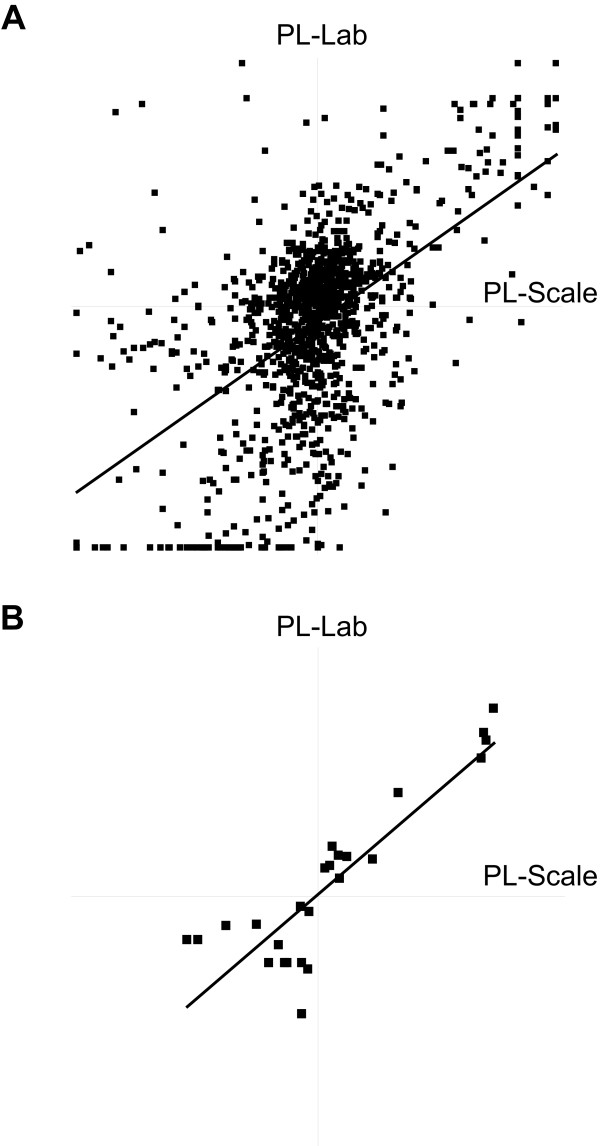
**Peptide ratios from two different infection methods**. Hemolymph was collected from infected and healthy 5-day old larvae. In (a), ratios of the 1207 peptides concomitantly quantified two different infection methods is shown. In (b), twenty-five proteins quantified with a minimum of 95% confidence are shown. All values are shown in natural log scale, relative to the control hemolymph (PL-Scale, x-axis, PL-Lab, y-axis). Linear regression is represented by the diagonal lines: (a) slope = 0.69, y-intercept = 0.28, R^2 ^= 0.29; (b) slope = 0.85, y-intercept = 0.041, R^2 ^= 0.76.

In order to test the response of worker larvae to *P. larvae *infection, we spray-inoculated a small section of comb containing one-day-old worker larvae with either (A) a homogenate of scale from natural infections of *P. larvae *(PL-Scale) or (B) a laboratory-cultured strain of *P. larvae *(NRRL B-3650, PL-Lab). We used two sources of *P. larvae *as we had no *a priori *knowledge regarding their relative pathogenicity. Four days post-infection we harvested hemolymph from 5-day larvae and compared the protein expression in the two infected conditions versus an uninfected control using a quantitative proteomics approach [11]. Using an ultra-high accuracy/resolution LTQ-OrbitrapXL, we identified a combined total 331 proteins (listed in Additional Files 1 and 2) with an estimated false discovery rate of 0.30%. Protein regulation differed between bees infected with the PL-Lab strain compared to the PL-Scale strain (Fig 1a, p < 0.0001) but among the 25 proteins quantified in both infections (Fig 1b) that were significantly different from control (p < 0.05), 40% (10) were higher in PL-Scale and 60% (15) were higher in PL-Lab. Thus, while there were differences between the inoculums, they were not consistently in one direction and so there was not enough evidence to reject the null hypothesis that regulation among these shared proteins differed between the two strains (Wilcoxon matched-pairs signed-ranked test, p = 0.87).

### Diseased honey bee larvae express higher levels of mitochondrial metabolic enzymes

**Figure 2:**
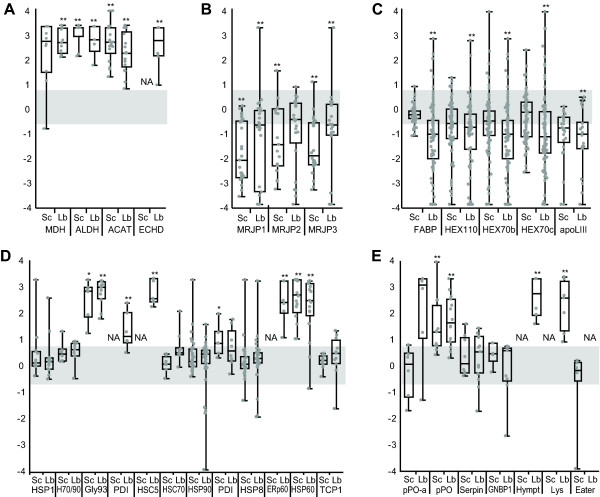
**American Foulbrood-induced changes in protein expression regulation in selected functional families**. Proteomes of honey bee hemolymph from larvae (5 days after hatching) infected using methods PL-Scale (Sc) or PL-Lab (Lb) were compared with healthy controls. Relative levels are expressed in the natural log scale (y-axis), with the level from uninfected bees defined at 0. Data points are relative peptide ratios pooled from 3 biological replicates, with the horizontal bar representing the median level of protein regulation. Those with the median beyond 2-fold (outside of the shaded box) and meets statistical significance, as calculated by the two-tailed Wilcoxon matched-pairs signed-ranked test are marked by a single (*, p < 0.05) or double (**, p < 0.01) asterisk. Selected proteins and functional families are shown: (a) highly regulated metabolic proteins, (b) major royal jelly proteins, (c) energy storage proteins, (d) protein folding chaperones, and (e) immunity-related proteins. NA = unquantifiable proteins. Protein abbreviations in alphabetical order (name, accession number): ACAT (acetyl-CoA acyltransferase, [GI:48097100]); ALDH (aldehyde dehydrogenase, [GI:66530423]); apoLIII (apolipophorin III, was "hypothetical protein", [GI:66557660]); Eater (a homolog identified by [10], [GI:110763407]); ECHD (enoyl-Coa hydratase, [GI:110773271]); ERp60 (a homlog of protein disulfide isomerase, [GI:66546657]); FABP (retinoid- and fatty acid binding protein, [GI:110758758]); Gly93 (glycoprotein 93, a homolog of HSP90, [GI:110758921]); GNBP1 (Gram-negative binding protein 1, [GI:110755978]); H70/90 (HSP70/90 organizing protein, [GI:110756123]); HEX110 (hexamerin 110, was "larval serum protein 2", [GI:110761029]); HEX70b (hexamerin 70b, [GI:58585148]); HEX70c (hexamerin 70c, was "hexamerin 2 beta", [GI:66549815]); HSC5 (heat shock protein cognate 5, [GI:66501507]); HSC70Cb (heat shock cognate 70Cb, [GI:66505007]); HSP1 (heat shock protein 1, [GI:110749824]); HSP60 (heat shock protein 60, [GI:66547450]); HSP8 (heat shock protein 8, [GI:66537940]); HSP90 (heat shock protein 90, [GI:66512625]); Hympt (hymenoptaecin, [GI:58585174]); Lys (lysozyme, [GI:66565246]); MDH (malate dehydrogenase, 66513092); MRJP1 (major royal jelly protein 1, [GI:58585098]); MRJP2 (major royal jelly protein 2, [GI:58585108]); MRJP3 (major royal jelly protein 3, [GI:58585142]); PDI (disulfide isomerase, [GI:110768510]); PDI (disulfide isomerase, [GI:66531851]); pPO (prophenoloxidase, [GI:58585196]); pPO-a (prophenoloxidase-activating factor, [GI:110758534]); Serpin (serine protease inhibitor 5, [GI:66566441]); TCP1 (a homlog of chaperonin, [GI:66560172]).

A total of 33 proteins, out of 179 quantified, were regulated by a magnitude of at least 2-fold for either one or both inoculums (p < 0.05 or p < 0.01, two-tailed, non-parametric Wilcoxon matched-pairs signed-ranked statistical test, full results listed in Additional File 3). Among the most up-regulated of all quantified proteins were several mitochondrial metabolic enzymes (Fig 2a). One malate dehydrogenase (MDH) [GI:66513092], for example, showed about a 14-fold increase by both infection methods (p < 0.01). This bee MDH is 67% identical to human mitochondrial MDH2 [GI:12804929], implying its direct participation in the mitochondrial matrix and the tricarboxylic acid (TCA) cycle, instead of the malate-aspartate shuttle that is carried out by human MDH1 [GI:66506786]. Further to this point, the levels of aspartate aminotransferase, another major enzyme of this shuttle, showed no change. An aldehyde dehydrogenase (ALDH) [GI:66530423], a homolog of the human mitochondrial isoform [GI:118504] was up-regulated 25-fold (p < 0.01) in PL-Lab-infected samples, and the same trend was observed in infection with PL-Scale, although it did not reach statistical significance (p < 0.1). Acetyl-CoA acyltransferase [GI:48097100], which participates in beta-oxidation and the mevalonate pathway, was significantly (p < 0.01) up-regulated at 14- and 9-fold in PL-Scale and PL-Lab, respectively.

### Infected larvae deplete their energy stores during infection

Clearly the metabolic capacity of larvae is undergoing a massive change in response to infection (Fig 2a), suggesting that concerted changes may also be occurring in their energy stores. Food proteins, which comprise a family called the major royal jelly proteins (MRJPs), are consistently depleted (Fig 2b), except in one case that was not significant at the p < 0.05 level. At the same time, 5 d larvae should be accumulating enormous levels of hexamerin (HEX) proteins in the hemolymph [11] as an amino acid source for later growth in the pupal stage. However, HEX110 [GI:110761029], HEX70b [GI:58585148] and HEX70c [GI:66549815] have a modest but significant (p < 0.01) 2- to 3-fold decrease under PL-Lab infection conditions (Fig 2c). Similar reductions were seen for two lipid carriers, retinoid- and fatty-acid binding protein [GI:110758758] and apolipophorin III [GI:66557660], while a putative neuropeptide Y (NPY) receptor [GI:110764421] that may regulate food intake was strongly up-regulated by the PL-Lab infection (p < 0.05)

### The protein-folding/quality control machinery is over-expressed in response to infection

Protein-folding chaperones and heat-shock proteins (HSPs) have been implicated in disease responses due to stress associated with tissue damage [13], with evidence that they also have roles in signal transduction in immune pathways [14]. Twenty-six molecular chaperones were detected in larval hemolymph, with many being up-regulated 3- to 20-fold in diseased larvae (Fig 2d). Among them are three proteins with multiple domains homologous to disulfide isomerases ([GI:110768510], [GI:66546657], [GI:66531851]), a 90 kDa heat shock protein HSP90 [GI:110758921], a 60 kDa heat shock protein HSP60 [GI:66547450] and a heat shock cognate 5 homolog [GI:66501507]. In human studies, heat shock proteins such as HSP60 have been repeatedly linked to macrophage activation [15,16]. Hemocytes, being somewhat similar to macrophages in their phagocytic capacity, have been noted to undergo morphological changes during AFB infection, while at the same time populations of other hemolymphic cells increase [17] so these effects may be linked with the HSP up-regulation observed here.

### Lysozyme and hymenoptaecin levels increase with bacterial challenge

We were able to identify four low molecular weight defense proteins: lysozyme [GI:66565246], hymenoptaecin [GI:58585174], apidaecin 22 [GI:58585226], and defensin [GI:58585176]. We observed a 13-fold increase of lysozyme in PL-Lab infections (p < 0.01) and a 16-fold increase of hymenoptaecin (Fig 2e) but there were too few peptides detected for apidaecin 22 and defensin to meet our criteria for quantitation (see Materials and Methods). Other immune factors that were identified but did not appear to be regulated by infection include a Gram-negative bacteria binding protein [GI:110755978], peptidoglycan recognition protein (PGRP) SA [GI:110765019] and PGRP SC2 [GI:66522804], suggesting that the response seen for lysozyme and hymenoptaecin is a specific response to *P. larvae *infection.

### ProPO expression and proteolytic activation are enhanced during infection

**Figure 3:**
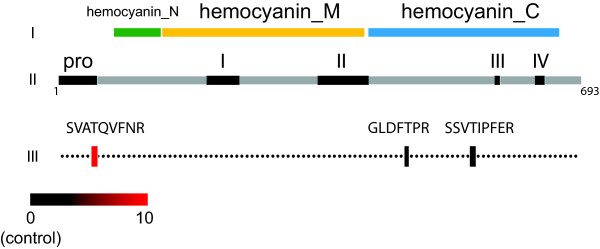
**Mass spectrometry-based peptide analysis for prophenoloxidase (proPO)**. Protein domains [22] of proPO are shown in row I. Notable regions [19] and protein length are shown in row II. Row III describes the average quantity of three peptides in infected larval hemolymph, represented by a color scale to depict fold-differences relative to healthy controls (black). Peptides used in averages and their statistical significances using the two-tailed, paired t-teset: n = 3 for SVATQVFNR (p < 0.05), n = 3 for GLDFTPR (p < 0.1), n = 5 for SSVTIPFER (p < 0.05). Averages were generated by considering values from both infection methods (PL-Scale and PL-Lab) together.

The melanization cascade, which leads to the encapsulation of infectious agents, is one of the most important defensive mechanisms of insect innate immunity. One of the central steps in this mechanism is the cleavage of proPO to PO, the active form of the monooxygenase. The PO enzyme, which is activated by proteolytic action, catalyses a key step in the synthesis of melanin and plays a crucial role in melanotic encapsulation of invaders [18]. We observed a 4-fold increase (p < 0.01) in the expression of proPO in PL-Scale-infected larvae and a 5-fold increase (p < 0.01) in PL-Lab-infected larvae (Fig 2e). The tryptic peptide SVATQVFNR, whose C-terminus is the predicted propeptide cleavage site [19], was elevated by about 10-fold compared to tryptic peptides found in the remainder of the protein (Fig 3). The higher ratios for this propeptide versus the other peptides of the protein suggest that the increased PO response during infection is largely due to the proteolytic activation of an existing pool of PO in the hemolymph and only partially attributable to up-regulated expression.

### Phenoloxidase activity is not found in the first two days of larval development, but increases sharply afterwards

**Figure 4:**
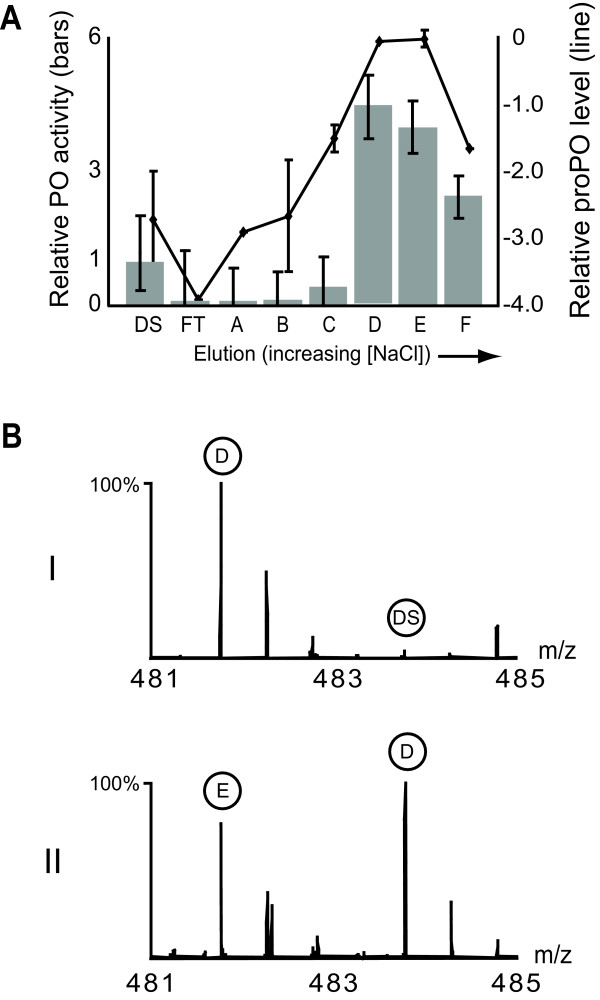
**Hemolymph fractionation**. (a) Hemolymph from fourth- and fifth-instar larvae was fractionated by strong anion exchange using a step gradient of increasing salt. Each fraction (A-F from 0.04 M to 0.24 M salt in 0.04 M increments, plus DS = desalted hemolymph, and FT = flowthrough) was normalized by protein concentration and was subjected to a PO assay (see Methods). Activity is represented by relative reaction rates to DS (left axis, bars). Using mass spectrometry, proPO levels were measured relative to the fraction containing the highest activity (Fraction D), shown on a natural log scale (N = 3). This was accomplished by averaging the ratio of at least two peptides for proPO in a differentially label mixture of peptides from Fraction F versus the remaining fractions. Error bars = 2 standard deviations. As an example in (b), two spectra of the differentially labeled (+28Da and +32Da) peptide FSDTIVPR is shown at a 1:1 mixture of peptides from (I) Fraction D and sample DS and (II) Fraction D and E.

Although PO is well-known for its activity against pathogens, there is little indication so far that its expression level affects the outcome of infection by *P. larvae*. Recent data from our group suggests that proPO levels correlate positively with age [11], but we had been unable to establish a full profile of proPO levels during the entire course of larval development due to the low absolute levels of expression. A PO activity assay [20], used to test for the oxygenation of monophenols to diphenols and diphenols to quinones [21], should be more sensitive than mass spectrometry and so was employed here to detect PO activity in developing, healthy larvae. PO activity was easily detected in crude hemolymph from fourth- to fifth-instar honey bee larvae but there are at least two gene products in the honey bee genome that could function in this assay based on domain comparisons [22]. To determine which of the two possible proteins is responsible for the PO activity in hemolymph, hemolymph from healthy, fourth and fifth instar larvae was fractionated by strong anion exchange chromatography and the PO activity in each fraction was correlated (Fig 4a) with the abundance of each protein [23]. The measurement of relative proPO levels was accomplished using differential isotopic labeling of peptides in each fraction, selecting one fraction as the reference to compare against the others (Fig 4b). The PO activity profile matched very closely to the levels of the gene product annotated as 'prophenoloxidase' [GI:58585196] across the chromatographic fractions and matched very poorly to HEX70b, which also has a putative phenoloxidase catalytic domain.

**Figure 5:**
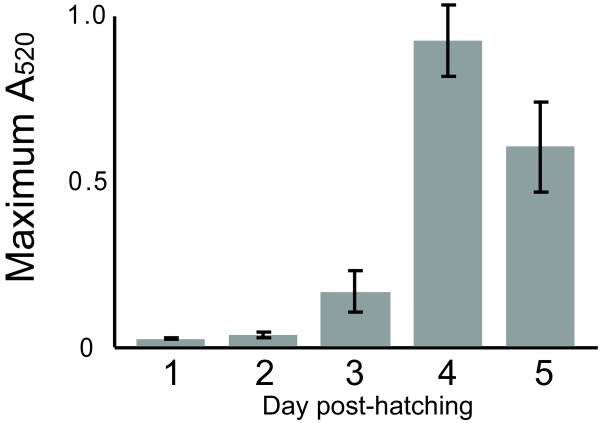
**Phenoloxidase activity assays**. Protein concentration-normalized hemolymph was collected over the first five days of larval development. The PO activity assay (see Methods) was conducted on the samples, where activity is represented here by the maximum A520 attained by the samples. All PO assay measurements were performed in triplicate. Error bars = 2 standard deviations.

Conceivably, older larvae can boost PO activity in response to infection, but could a lack of PO activity in the early larval stages explain the susceptibility of young larvae to *P. larvae *infection? Cell-free hemolymph was extracted from healthy larvae one to five days after eclosion and tested for PO activity and, indeed, there was no detectable activity in the first two days of development, with some activity detected in day three and substantial activity thereafter (Fig 5).

## Discussion

Here we have used a quantitative proteomics approach to compare the proteomes of healthy and *P. larvae*-infected *A. mellifera *larvae, leading to the discovery that the infected state is associated with an elevated expression of immunity proteins, chaperones, certain metabolic proteins with an accelerated consumption of energy stores. One particular immune factor, proPO, was particularly up-regulated in response to infection. Intriguingly, the activity level of this enzyme during development of larvae appears to correlate very tightly with susceptibility of the larvae to infection. Our data support a model where the host larva responds to infection not only by producing proteins that can fight the infection directly, but also by engaging its metabolic pathways and energy resources required to support the effort.

The observed depletion of energy stores in the form of MRJPs, hexamerins and lipid transporters suggest that the observed up-regulation of metabolic enzymes is, at least in part, tied to energy production. In larvae infected with *E. coli*, which is not a natural pathogen of bees, MRJP-1, MRJP-7/MRJP-2 were mildly lowered in expression compared to the mock-infected control [12]. The negative correlation between energy availability and infection survival has also been observed in other insects such as the butterfly *Pieris rapa *[24] and the bumblebee *Bombus terrestris *[25]. In further support of the increased energy demands of the infected state, the putative bee neuropeptide Y receptor was also up-regulated in infected larvae. While the ligand for this receptor in bees remains unknown, the receptor and its cognate ligand in mammals control feeding and appetite in mammals [26].

The most obvious class of proteins expected to increase in response to infection are those involved in the innate immune response. Lysozyme's primary known function is to degrade the peptidoglycan shell of Gram-positive bacteria [27] and is therefore expected to have a significant role in inhibiting *P. larvae*. Interestingly, the C-type lysozyme [GI:110762174] that has been previously shown to be up-regulated upon infection [10] is not the lysozyme we have identified here, which is also known as the destabilase-lysozyme [GI: 6656246]. Because these two forms are drastically different (e.g., the best-matched region is only 50 amino acids long and shares only 20% sequence identity), it is clearly not a case of the peptides identified by MS/MS being shared by both enzymes. The 13- to 16-fold up-regulation of destabilase-lysozyme suggests that it can be important in host defense, which is also supported by the observation that its homolog has antimicrobial activity in the medicinal leech *Hirudo medicinalis *[28]. The AMPs comprise another humoral-based defense mechanism, killing Gram-negative and positive bacteria alike [12,29]; many of them work by forming pores in the bacterial cell wall. Among those known in bees, we were only able to quantify hymenoptaecin in the hemolymph, and its dramatic up-regulation suggests that it plays a crucial role in defending against *P. larvae*, a conclusion that is supported by other reports [10,12]. Conspicuously absent in our data, however, are defensin and abaecin, which have both been implicated in the larval response to *P. larvae *[6-10]. Although we detected peptides from defensin, the signal:noise ratios in the MS^1 ^spectra were not high enough to allow accurate quantitation; no abaecin peptides were detected. Our inability to detect these two AMPs with sufficient signal suggests that their concentration is likely much lower than hymenoptaecin, which is confirmed by a recent one-dimensional (1D) gel electrophoresis study of larval hemolymph [12].

The consistent up-regulation of proPO in both infection methods is in agreement with the well-characterized antimicrobial activity of this enzyme. The ability of larvae to employ melanization as a defense mechanism has been questioned because the proPO levels are low compared to adults, to the point of being undetectable on a stained 1D gel [12]. In our own experiments, even with ion-exchange fractionation prior to LC-MS/MS analysis on an LTQ-OrbitrapXL, one of the most sensitive systems available, it was difficult to detect throughout most of larval development except for the oldest samples (5 d post-hatching) [11]. However, we are clearly able to detect PO activity as early as three days after hatching, where the absence of activity in the earlier timepoints (days 1 and 2) precisely match the period of maximum susceptibility of the larvae to AFB [1,30]. Thus, our data argue that older larvae have significant levels of PO and that they are indeed capable of utilizing the PO pathway to fight infection.

## Conclusion

The larval stage of a honey bee represents a unique system for applying proteomics to probe host-pathogen interactions. Unlike most other systems, proteins in larvae not only play major roles in immune defense but also constitute one of their primary stores of energy. Studying such a response in most other systems with more conventional energy reserves (*e.g*., lipids) would necessitate a wide variety of tools in order to monitor energy usage, immune factor production and metabolic flux all at the same time. By monitoring all these aspects simultaneously, our data clearly demonstrate that host defense against bacterial challenge is a concerted response involving proteins that kill the microbes directly, as well as metabolic and cell/protein repair enzymes that indirectly support this defensive effort. By using proteomics techniques on this unique model organism where immunity and protein energy flux are tightly coupled, we have been able to build a more comprehensive picture of the insect innate immune response.

## Methods

### Honey bees and infection experiments

All infection experiments were conducted at Beaverlodge, AB, Canada during July and August of 2005. Three five-frame nucleus colonies ('nucs') were prepared with three frames of bees and open brood and with newly-mated sister queens. In each nuc, 100 by 100 mm patches of first instar larvae were selected and sprayed with 20 mL of one of the following: 1) a 6.0E+06 spores/mL suspension of spores isolated from naturally occurring AFB 'scale' collected in 2004 (PL-Scale), 2) a 4.4E+06 spores/mL suspension of spores from NRRL B-3650 (PL-Lab), a virulent laboratory strain of *P. larvae *(courtesy of Jay Evans), and 3) phosphate-buffered saline (PBS).

### Sample collection and processing for MS

Four days after infection larvae (estimated to be in late fourth or fifth instar) within each marked square (PL-Scale, PL-Lab and control) were extracted using soft forceps (Bioquip, Rancho Dominguez, CA) and bled as described [11]. Hemolymph was processed as described for larvae in [31]. In each case, we compared 20 μg of protein from infected hemolymph with the control by differential labeling of tryptic peptides using light (C^1^H_2_O) and heavy (C^2^H_2_O) isotopologs of formaldehyde prior to analysis by liquid chromatography-tandem mass spectrometry (LC-MS/MS) using a linear trapping quadrupole-OrbitrapXL exactly as described [11].

### MS data analysis

Raw data processing to arrive at peptide ion volume ratios was performed exactly as described [11]. Data for each infection method were pooled, and proteins with five or more quantified peptides from any one or all of the replicates were considered quantified. This approach operates on the underlying assumption that each peptide ratio is a technical replicate, which is different from most publications where averages are made at the protein, not the peptide level. The conventional method implies that a protein quantified from averaging over a large number of quantified peptides has the same statistical power than one quantified with the bare minimum. To circumvent this disadvantage, we took single peptides from three biological replicates as individual data points, which reasonably accounts for the greater statistical power afforded by well-detected proteins with many quantified peptides. The average level of protein regulation is represented by the median peptide ratio. We employed the two-tailed Wilcoxon matched-pairs signed-ranked test on these proteins using Analyse-It (v2.12, http://www.analyse-it.com/), with peptides as data points to assess whether the expression level of each protein was significantly changed by infection at 95% and 99% confidence [32]. For proteins with more than 100 peptides, the 100 most intense [M+nH]^n+ ^ions (heavy and light combined, n ≥ 2) were selected for analysis. The same test was used on peptides quantified in both infection methods to assess whether the two methods yielded the same effects on protein expression. Experimentally or bioinformatically-inferred evidence of protein functions and names discussed throughout this report is provided in Additional File 4.

### Hemolymph collection for PO activity assay

We collected honey bee larvae and estimated their age in days by size. Animals at each age were pooled to collect at least 8 μL of hemolymph per replicate for three replicates – the number of larvae required varied from approximately 150 for the very young larvae, and 2–5 for the oldest larvae tested (five days old); 18 fourth to fifth instar larvae were pooled and used for the fractionation experiment and processed as described for larvae in [31]. Protein concentrations were assayed by Coomassie Plus (except in the protein fractionation experiment, see below) and were normalized across all samples using Assay Buffer (20 mM Tris-HCl, pH 8).

### Hemolymph fractionation

Hemolymph was desalted using a mini Zeba column (Pierce) according to the manufacturer's instructions. Of the 75 μL total volume, 25 μL was reserved for MS analysis. The remainder was applied to a mini strong anion exchange column (Pierce), and washed with 100 μL of Assay Buffer between step-elutions of a 10-step sodium chloride gradient prepared in Assay Buffer: 0.04, 0.08, 0.12, 0.16, 0.20, 0.24, 0.28, 0.32, 0.36, 0.40, 0.50, 1.0, 2.0 M. Protein concentrations of all the fractions, including the flow-through and desalted hemolymph, were estimated by absorbance at 280 nm. Fractions eluted from 0.28 M or higher salt had negligible amounts of protein and were not further analyzed. The protein concentration of the other fractions was equalized using Assay Buffer. For each fraction we then measured the PO activity using an enzyme assay (see below) and levels of each protein relative to the 0.16 M NaCl fraction. At least 2 peptides of PO were used for calculating the average PO level in each fraction. In cases where peptide ratios were above 50-fold and likely beyond the linear dynamic range of the ratio measurements, the high ratios were arbitrarily given the same value as the next highest ratio value below 50-fold.

### In solution phenoloxidase assay

Conducted as described [20], substrate (8 μL of 5 mM 4-methylcatechol (Sigma) and 8 μL of 40 mM 4-hydroxyproline ethyl ester) was added to 8 μL of hemolymph to start the reaction, except for the experiment with larvae of different ages where the hemolymph:subtrate ratio was 4:1. Absorbance readings at 520 nm were taken immediately using a Nanodrop spectrophotometer (ND-1000, ThermoFisher Scientific) to calculate the initial reaction rate (ΔA_520_/ΔT). For larval aging experiments, instead of the rate, the maximum A_520 _value was recorded after the highest level was reached in approximately 40 min.

## Authors' contributions

APM and SFP designed and performed the colony infection experiments. APM and LJF collected the hemolymph samples in Beaverlodge. QWTC and LJF designed the proteomic analyses and wrote all the scripts used in the data analysis. QWTC performed all the proteomic, biochemical and bioinformatic analyses. QWTC and LJF wrote the initial version of the manuscript.

## Supplementary Material

Additional file 1 **This file contains the list of proteins considered identified by mass spectrometry-based sequencing and the peptide sequences of each protein.** Protein accession numbers preceded by "999" are proteins that have been falsely discovered by matches reversed peptide sequences. Click here for file

Additional file 2 This file contains the list of quantified peptides and their relative expression values. Click here for file

Additional file 3 This file contains the list of proteins, their median averaged values based on peptide relative expression. Click here for file

Additional file 4 This file contains the list of proteins discussed in the paper with direct mention of their known or putative function, and the evidence or resource for this information. Click here for file
